# Acute gastric volvulus in a patient with trisomy 21

**DOI:** 10.1186/s40792-014-0005-1

**Published:** 2015-01-16

**Authors:** Kota Arima, Daisuke Hashimoto, Noboru Takata, Yasuro Doi, Ichiro Yoshinaka, Kazunori Harada, Hideo Baba

**Affiliations:** Department of Surgery, Amakusa Regional Medical Center, 854-1 Jikiba, Kameba-machi, Amakusa, Kumamoto 863-0046 Japan; Department of Gastroenterological Surgery, Kumamoto University Graduate School of Medical Sciences, 1-1-1 Honjo, Chuo-ku, Kumamoto 860-8556 Japan

**Keywords:** Gastric volvulus, Down syndrome, Emergency operation

## Abstract

Acute gastric volvulus is a torsion of the stomach by more than 180° and a life-threatening condition. We present a 50-year-old male patient with acute abdominal pain who has Down syndrome/trisomy 21. Computed tomography showed a significant distended stomach with features of a severe gastric volvulus. Emergency operation in form of reduction and gastropexy was performed. We are not aware of any similar cases published in the English literature, where as gastric volvulus occurred in a patient with Down syndrome.

## Background

Acute gastric volvulus is a rare but potentially life-threatening condition due to possible gastric necrosis. The cause of this disease is surmised to the insufficient fixation by intraperitoneal visceral ligaments. There have been a number of case reports on volvulus cases in the past; however, a case of it with a chromosomal abnormality as one of the possible major causes has never published [1-5]. We herein report a rare case of acute severe gastric volvulus that occurred in a patient with Down syndrome.

## Case presentation

**Figure 1:**
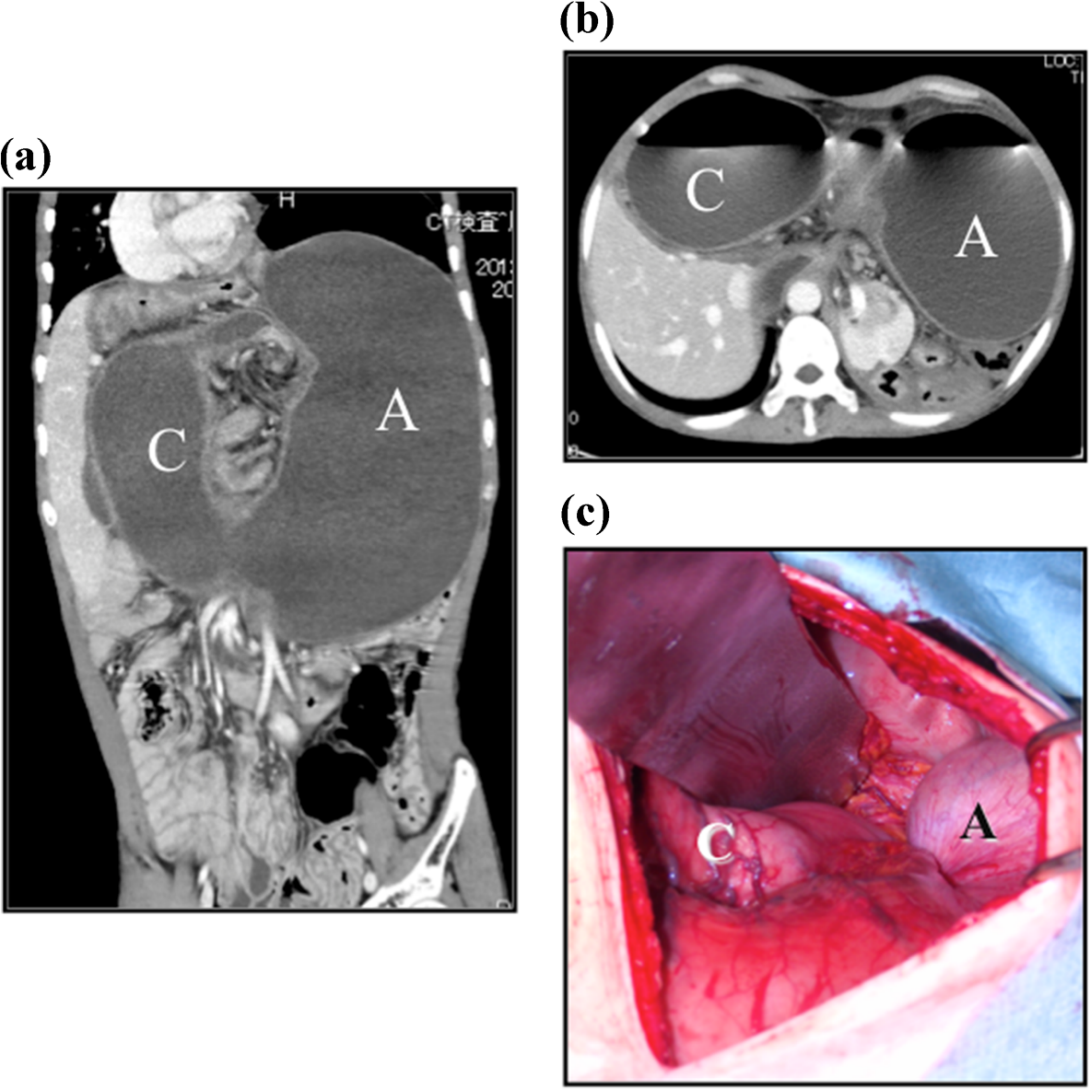
**Preoperative and operative findings.** Axial **(a)** and coronal view **(b)** of the enhanced computed tomography revealed dilatation and volvulus of the stomach. Laparotomy **(c)** showed a distended stomach rotated 180° around mesoaxis at the cardia and pylorus. A, antrum; C, cardia.

A 50-year-old male patient, with Down syndrome/trisomy 21 derived from a 14/21 Robertsonian translocation, was presented at our hospital who suffered from severe abdominal pain. He showed a severe intellectual disability and had a megacolon. Abdominal examination revealed abdominal distention but did not indicate any muscular defense. Laboratory investigations showed elevations in the white blood cell count (16,900 cells/mm^3^), neutrophil fraction (91.3%), and C-reactive protein (7.3 mg/dl). Contrast-enhanced computed tomography (CT) showed a significant distended and twisted stomach (Figure 1a,b), suggesting a diagnosis of mesenteroaxial gastric volvulus.

**Figure 2:**
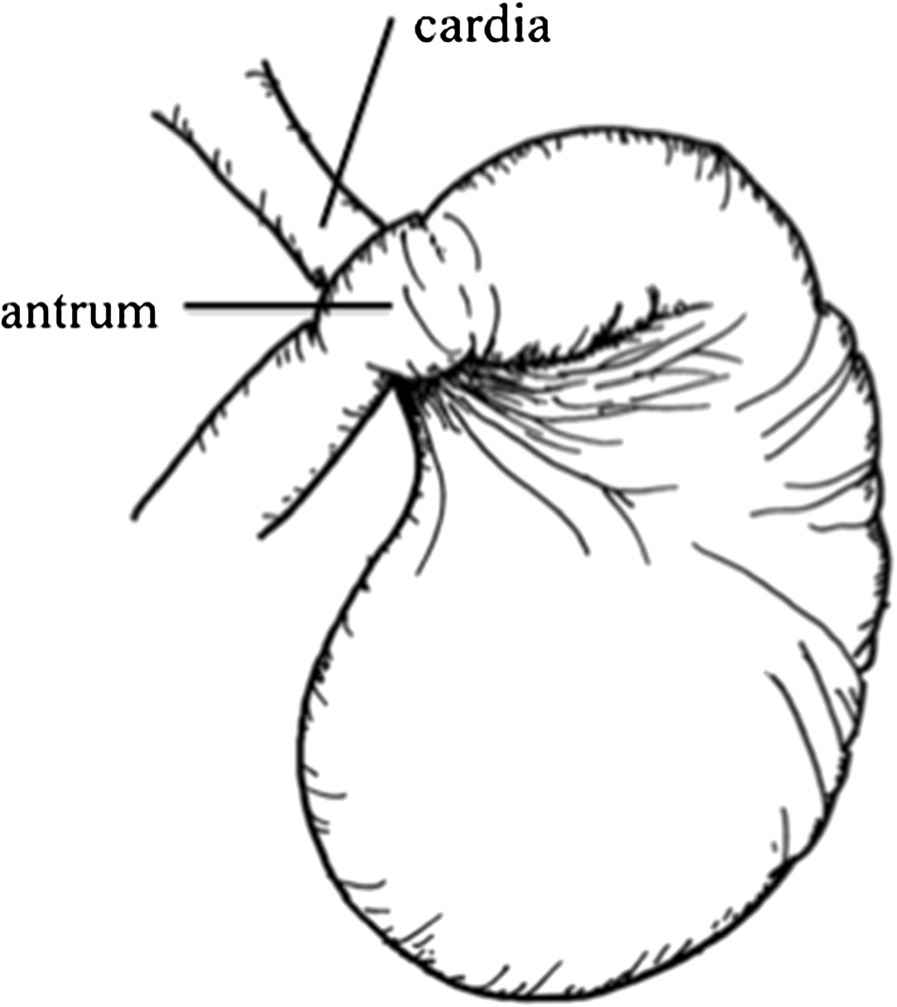
**Preoperative scheme.** A scheme shows the state of the stomach.

**Figure 3:**
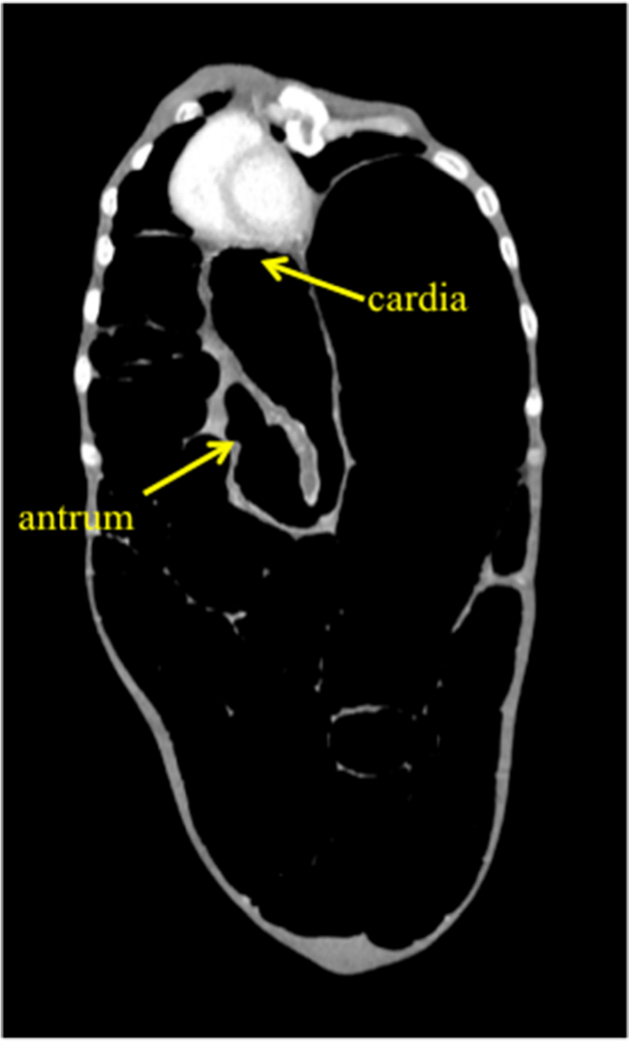
**Postoperative findings.** The coronal view of the enhanced CT revealed the recovery of gastric volvulus after surgery.

As a nasogastric tube or a gastroscope was unable to reach the stomach, it was opted to immediately perform an emergency operation. Due to the severe distended stomach, adequate intra-abdominal space was not available so that laparoscopic operation was no option for this patient as report in other cases [6,7]. Laparotomy showed a large, distended, and twisted stomach (Figure 1c and 2). The stomach was successfully repositioned, and gastropexy in combination with the Coffey method was performed (Figure 3).

### Discussion

Gastric volvulus was reported to be more likely to be complicated by wandering spleen, diaphragmatic eventration, and malrotation of the intestine [8-11]. Even though major gastrointestinal malformation, such as congenital esophageal atresia, has been reported to occur in Down syndrome patients, we are not aware of cases of gastric volvulus published in the English literature that occurred in a patient with Down syndrome [12,13]. Primary gastric volvulus is related to the lack of fixation by the ligaments which exist between a stomach and other organs. These gastric ligaments were significantly extended in this patient, and this malformation was most likely a major cause of gastric volvulus. If the gastric ligaments are extended or absent, the stomach may rotate easily. As they were significantly extended in this patient, this malformation was most likely a major cause of gastric volvulus in our patient. Furthermore, even though there are some reports of deficiency of the gastric ligaments, there is no report about possible reasons of this deficiency and extension of it [11,14,15]. It is therefore important to be aware of the possible occurrence of gastric volvulus in the patients with trisomy 21.

## Conclusions

Gastric volvulus is a rare but life-threatening condition unless diagnosing and treating rapidly. We could save the patient of the gastric volvulus due to early diagnosis and surgery.

## Consent

Written informed consent was obtained from the patient for publication of this case report and any accompanying images. A copy of the written consent is available for review by the Editor in Chief of this journal.

## Abbreviations

CT: computed tomography
